# Hyponatremia Among Elderly Hospitalized Patients: An Observational Study

**DOI:** 10.7759/cureus.67632

**Published:** 2024-08-23

**Authors:** Sai Priya Ande, Shubhangi Kanitkar, Akshata Borle, Muskaan Ahlawat

**Affiliations:** 1 Medicine, Dr. D.Y. Patil Medical College, Hospital and Research Centre, Dr. D.Y. Patil Vidyapeeth (Deemed to be University) Pimpri, Pune, IND

**Keywords:** volume status, serum sodium levels, electrolyte imbalance, geriatric medicine, hyponatremia

## Abstract

Aim

The aim of this study is to analyze the demographic distribution (age and gender distribution), presenting symptoms, and evaluate the underlying etiology of hyponatremia among the study population. The presence of comorbidities and the volume status (hypovolemia, euvolemia, or hypervolemia) of elderly hyponatremic patients with varying severity of hyponatremia were assessed.

Methods

This cross-sectional, observational study was conducted in Dr. D. Y. Patil Hospital and Research Centre, Pune, India. After approval from the Institutional Ethics Sub-Committee (approval number: IESC/PGS/2022/09), it was conducted during the period between September 2022 and June 2024. The minimum sample size was calculated to be 96 with a confidence interval of 95% using WINIPEPI software (version 11.38). The lab values of serum sodium of all patients aged above 60 years admitted in wards and intensive care units (ICUs) were studied. Out of these hyponatremic patients, a sample size of 100 patients was randomly selected. Patients above 60 years and the patients who were on diuretic therapy were excluded from the study.

Results

The study included 100 elderly patients with a mean age of 73.25 ± 7.03 years, ranging from 64 to 86 years. Males predominated (63%), and severe hyponatremia (<125 mEq/L) was the most common, affecting 61% of patients. Generalized weakness (22%) and disorientation (17%) were the most frequently reported symptoms. Post-operative conditions (13%) and gastroenteritis (10%) were the leading causes. Most participants had no comorbidities (53%). Hypovolemia was present in 67% and euvolemia in 29% of the study subjects. Among hypovolemic patients, severe hyponatremia was present in 83.5% of patients.

Conclusion

This study highlights the significant burden of severe hyponatremia among elderly patients, particularly in male subjects and those with hypovolemia. Majority of the participants did not have any comorbidities. Additionally, the study emphasizes the need for heightened clinical vigilance in elderly patients presenting with generalized weakness and disorientation, as these were the most common symptoms associated with hyponatremia. The identification of post-operative conditions and gastroenteritis as leading causes further supports the need for comprehensive management strategies in elderly inpatients to prevent the occurrence and complications of hyponatremia.

## Introduction

Hyponatremia, characterized by a serum sodium concentration below 135 mmol/L, is the most common electrolyte disturbance encountered in clinical practice, particularly among elderly patients. This condition is frequently seen in the elderly due to age-related physiological changes, increased prevalence of comorbidities, and the use of multiple medications [1]. Hyponatremia can significantly impact the health and quality of life of older adults, leading to increased morbidity, mortality, and healthcare costs [2].

Hyponatremia can be classified into three main types based on volume status: hypovolemic, euvolemic, and hypervolemic. Hypovolemic hyponatremia is typically caused by losses of sodium and water, often due to conditions such as gastrointestinal losses or diuretic use. Euvolemic hyponatremia is commonly associated with the syndrome of inappropriate antidiuretic hormone secretion (SIADH), often seen in conditions such as malignancies, pulmonary diseases, and central nervous system disorders. Hypervolemic hyponatremia usually occurs in the context of conditions like heart failure, liver cirrhosis, and nephrotic syndrome, where there is an overall increase in body water relative to sodium [3].

The pathophysiology of hyponatremia in the elderly involves impaired renal water excretion, increased sensitivity to vasopressin, and altered thirst mechanisms. Additionally, the elderly are more susceptible to medications that can alter sodium and water balance, such as thiazide diuretics, antidepressants, and antiepileptics [4]. Chronic conditions common in the elderly, such as heart failure and chronic kidney disease, further contribute to the risk of developing hyponatremia [5].

The prevalence of hyponatremia in the elderly varies, but studies suggest it can affect up to 18% of elderly hospitalized patients, highlighting the need for heightened awareness and proactive management in this population [6]. In India, studies suggest a prevalence of hyponatremia among hospitalized elderly patients ranging from 18% to 29%, with higher rates observed in patients with comorbid conditions such as heart failure and chronic kidney disease [7]. Hyponatremia in elderly patients in India is associated with increased hospital stay, higher rates of complications, and greater mortality [8].

Treatment options for hyponatremia vary depending on the underlying cause and severity of the condition. In hypovolemic hyponatremia, the primary approach is volume replacement with isotonic saline. For euvolemic and hypervolemic hyponatremia, fluid restriction, vasopressin receptor antagonists, and addressing the underlying condition are key strategies. Monitoring and careful correction of sodium levels are crucial to avoid complications such as osmotic demyelination syndrome [9]. The present study endeavors to examine hyponatremia in the elderly, aiming to comprehend the symptoms, underlying etiology, presence of comorbidities, and volume status at the time of presentation.

## Materials and methods

Study design

The present study was a cross-sectional, observational study conducted at Dr. D. Y. Patil Medical College, Hospital, and Research Centre, Pimpri, Pune. The study period extended from September 2022 to June 2024. Approval was taken from the Institutional Ethics Sub-Committee (approval number: IESC/PGS/2022/09) before commencing the study. Written and informed consent was obtained from all patients. The patients were informed regarding the purpose, procedures, risks, and benefits of the study in their own vernacular language. The data was analyzed with an Excel spreadsheet and with the help of Statistical Product and Service Solutions (SPSS, version 20.0; IBM SPSS Statistics for Windows, Armonk, NY).

Inclusion Criteria

All patients >60 years of age with at least two serum sodium values <135 mEq/L admitted in wards and intensive care units (ICUs) were included.

Exclusion Criteria

All patients with <60 years of age and those who were treated with diuretics were excluded.

Sample Size

The lab values of serum sodium of all patients aged above 60 years admitted in wards and intensive care units (ICUs) were studied. Out of these hyponatremic patients, a sample size of 100 patients was randomly selected. Considering the proportion of elderly with hyponatremia from the study "Hyponatremia in Elderly In-Patients" as 47.9%, with a confidence interval (CI) of 95% and an acceptable difference of 10%, the calculated sample size was 96 [10]. Software used was WINPEPI, version 11.38 [10].

Data Collection Technique

In the hospital, as a routine, serum electrolytes were done, and the records were followed up for patients with hyponatremia and values repeated for confirmation. A standard proforma was used to record a detailed history of present complaints, past history, including diabetes mellitus, systemic hypertension, ischemic heart disease, neurological, chronic kidney disease/renal disease, and endocrine problems. A detailed drug history was recorded. Treatment received was noted, and the outcome of the patient was recorded.

## Results

**Figure 1:**
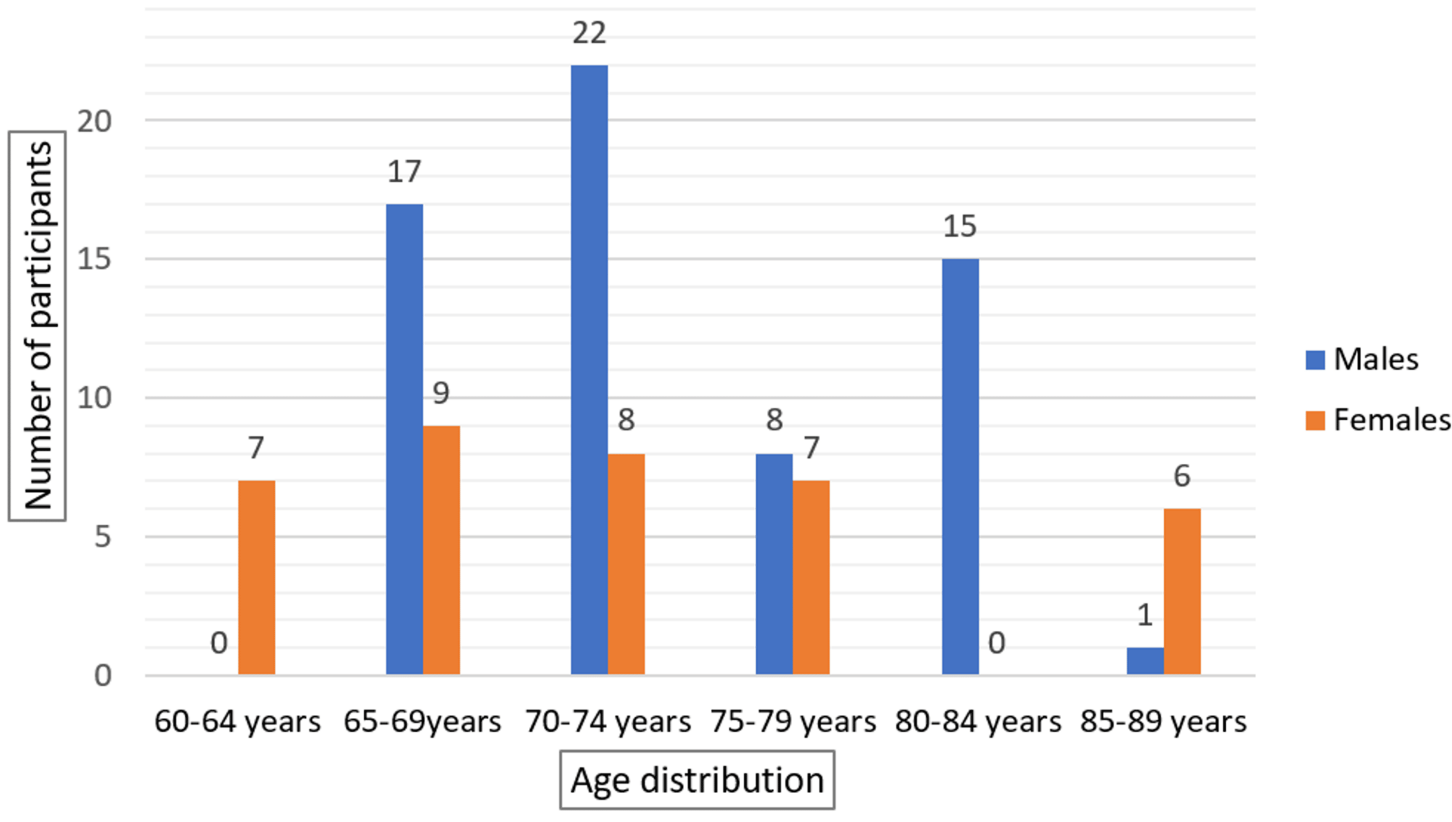
Age and Gender Distribution of Elderly Patients With Hyponatremia

The study included 100 elderly patients with a mean age of 73.25 ± 7.03 years (range: 64-86 years). Males comprised 63% of the participants and females 37%, with a male-to-female ratio of approximately 2:1. Most male participants were aged 70-74 years, while most female participants were aged 65-69 years (Figure 1).

**Figure 2:**
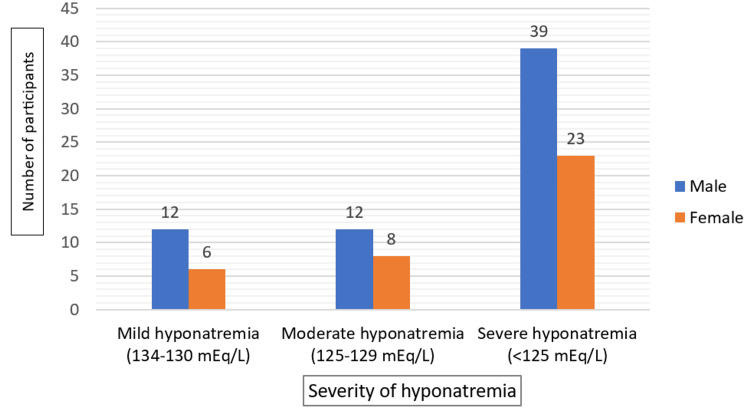
Gender Distribution Across Severity Levels of Hyponatremia in Elderly Patients

Severe hyponatremia (<125 mEq/L) was the most common, affecting 61% of patients (39 males and 23 females) (Figure 2). Moderate hyponatremia (125-130 mEq/L) and mild hyponatremia (130-135 mEq/L) were less prevalent.

**Figure 3:**
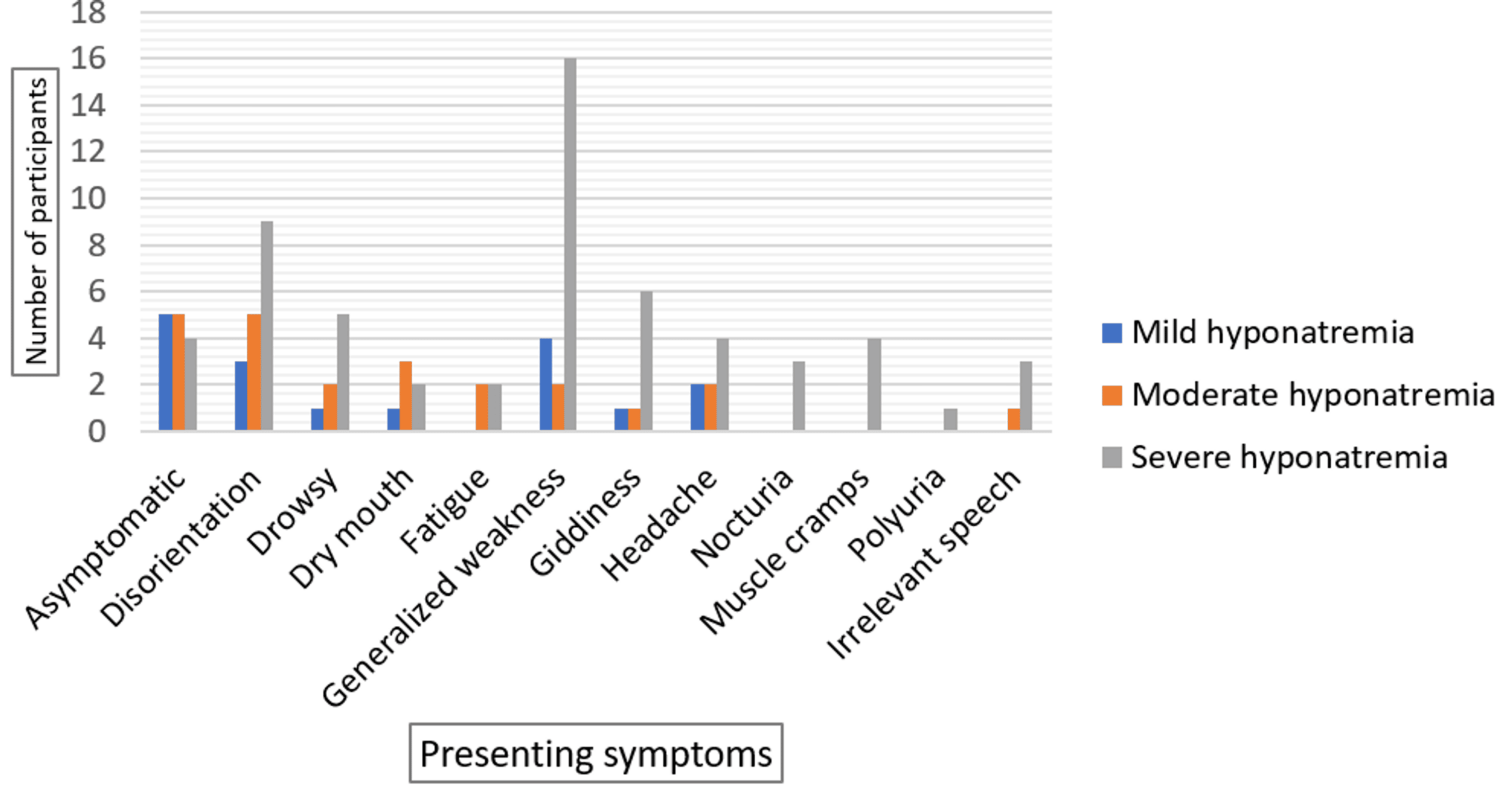
Symptom Distribution by Severity of Hyponatremia in Elderly Patients

Generalized weakness (22%) and disorientation (17%) were the most frequently reported symptoms, while 14% of the study population were asymptomatic at presentation. Other symptoms included giddiness, headache, drowsiness, dry mouth, fatigue, nocturia, irrelevant speech, muscle cramps, and polyuria (Table 1). Among patients with severe hyponatremia, generalized weakness, disorientation, and giddiness were the most common symptoms (Figure 3).

**Table 1:** Frequency of Symptoms in Elderly Patients With Hyponatremia

Symptom	Percentage (%)
Generalized weakness	22%
Disorientation	17%
Asymptomatic	14%
Giddiness	8%
Headache	8%
Drowsy	8%
Dry mouth	6%
Fatigue	4%
Nocturia	4%
Irrelevant speech	4%
Muscle cramps	4%
Polyuria	1%

The most common underlying causes of hyponatremia were post-operative conditions (13%) and gastroenteritis (10%). Other causes included urinary tract infection (UTI) (8%), poor intake (8%), cancer (8%), tuberculosis (7%), dilated cardiomyopathy (DCM) (6%), heart failure (7%), heat stroke (7%), lower respiratory tract infection (LRTI) (5%), and drug-induced hyponatremia (4%). Multifactorial causes were present in 17% of cases.

**Figure 4:**
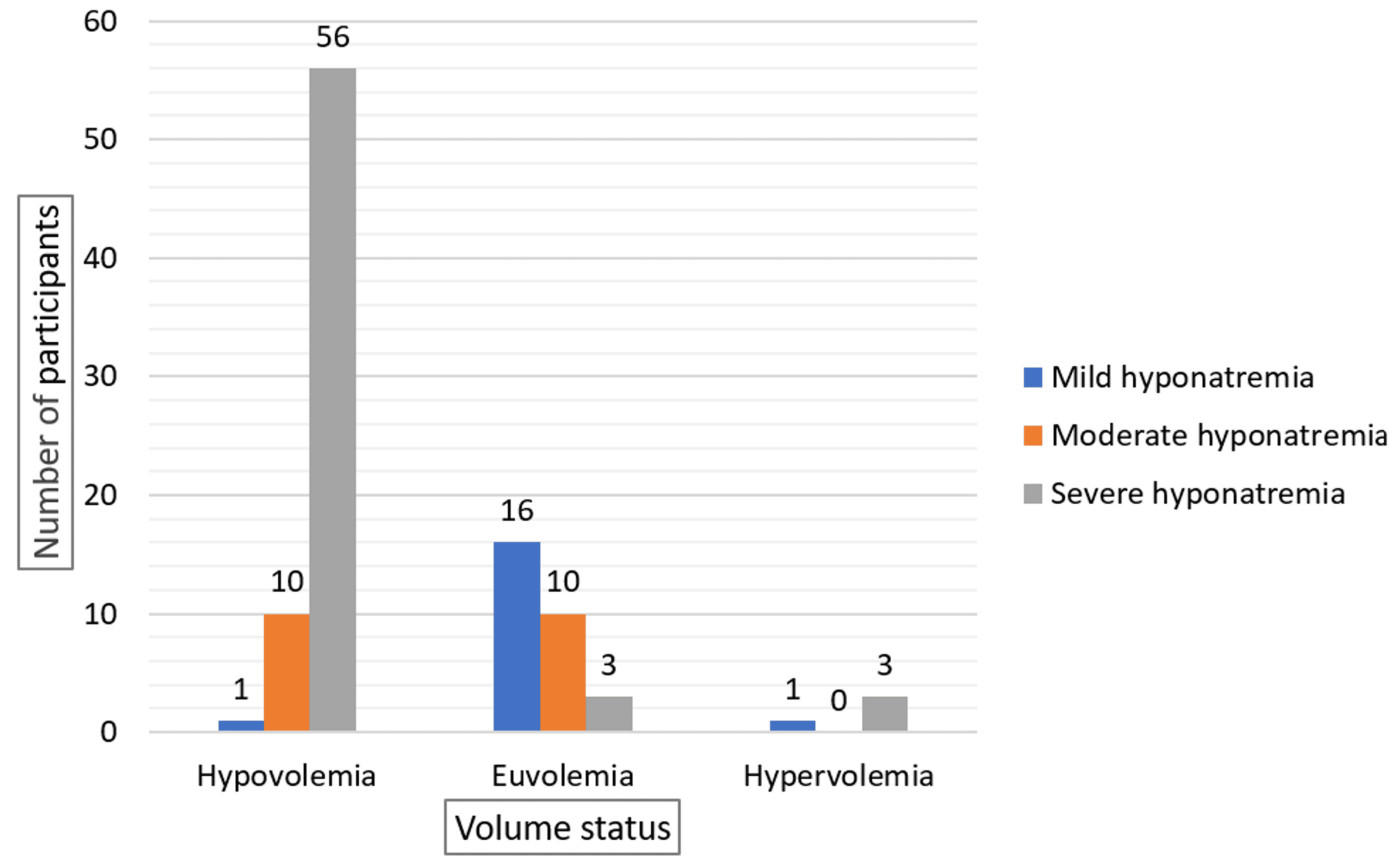
Volume Status Distribution by Severity of Hyponatremia in Elderly Patients

Hypovolemia was predominantly associated with severe hyponatremia (56%), while euvolemia was more balanced, with mild (16%) and moderate (10%) cases being more common. Hypervolemia was less common overall, while hypovolemia was the most prevalent among the study population (Figure 4).

**Figure 5:**
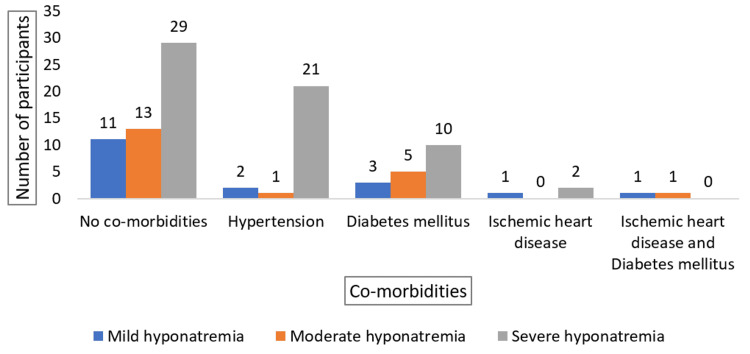
Distribution of Comorbidities by Severity of Hyponatremia in Elderly Patients

The highest prevalence of severe hyponatremia is observed in patients with no comorbidities and hypertension, while mild and moderate hyponatremia are relatively less common across all comorbidities. Notably, severe hyponatremia is significantly higher in patients with hypertension compared to other comorbidities (Figure 5).

## Discussion

An aged patient is more susceptible to hyponatremia due to a number of factors, including decreased glomerular filtration rate, diminished kidney function, increased production of antidiuretic hormone (ADH) in response to a particular osmotic stimulation, numerous medications they take, and coexisting disorders. The current study provides important insights into the clinical characteristics, underlying causes, and volume status of elderly patients with hyponatremia. The mean age of the study population was 73.25 ± 7.03 years, which is consistent with the mean age of 72 years reported in a hospital-based study by Rao et al. [11]. The male predominance (63%) observed in our study is also comparable to findings by Jain and Nandy, who reported a male-to-female ratio of 1:0.96 [12].

Generalized weakness (22%) and disorientation (17%) were the most frequent symptoms encountered, while 14% of subjects were asymptomatic at presentation. This symptom profile is similar to that reported by Bhattacharjee et al., where altered sensorium was the most common symptom [13].

In our study, 61% of patients had severe hyponatremia. This finding is in line with Bhattacharjee's study, which reported higher mortality rates with increasing severity of hyponatremia [13]. Post-operative conditions were the most common etiology (13%), followed by gastroenteritis (10%). This differs from other studies where heart failure and chronic liver disease were more prevalent causes. For example, Saeed et al. found that renal disorders, liver disorders, and congestive heart failure were significant causes of hyponatremia, which highlights the variation in etiological factors and underscores the need for individualized patient assessments and tailored management plans [14].

Comorbidities were present in 47% of the study population, with hypertension (24%) and diabetes (18%) being the most common. This prevalence is lower than that reported by Jain and Nandy, where hypertension and diabetes were more common [12]. 

Hypovolemic hyponatremia was the most prevalent with majority of patients with severe hyponatremia having hypovolemic hyponatremia. However, Baji and Borkar had noted a higher prevalence of euvolemic hyponatremia, particularly in patients with conditions affecting fluid regulation [15]. The limitations of the study are mentioned in Table 2.

**Table 2:** Limitations of the Study

Limitations	Explanation
Sample size	The study's sample size, although calculated to be statistically sufficient, is relatively small (100 patients) and may not fully capture the variability and diversity of the elderly population with hyponatremia.
Selection bias	Patients treated with diuretics were excluded from the study. Since diuretics are commonly used in the elderly, especially those with heart failure or hypertension, this exclusion may introduce a selection bias and affect the study's findings.
Cross-sectional design	The cross-sectional design of the study limits the ability to establish causal relationships between the observed variables. Longitudinal studies would be more effective in determining causality and the temporal sequence of events leading to hyponatremia.
Lack of long-term follow-up	The study does not include long-term follow-up of patients to assess outcomes such as recurrence of hyponatremia, long-term morbidity, or mortality. This limits the understanding of the long-term impact of hyponatremia in the elderly.

In conclusion, our study emphasizes the importance of early recognition and management of hyponatremia in the elderly, particularly focusing on presenting symptoms and volume status. While comorbidities did not show a significant correlation with hyponatremia severity, routine monitoring and a comprehensive approach to treatment are essential for improving patient outcomes. Further research is needed to explore the underlying mechanisms and develop targeted strategies for managing hyponatremia in this vulnerable population. The variation in etiological factors highlighted in this study and others, such as those by Saeed et al. and Baji and Borkar, underscores the necessity for tailored management plans to address the diverse causes of hyponatremia in elderly patients [14,15].

## Conclusions

This study highlights the complexity and vulnerability of elderly patients to hyponatremia, influenced by age-related physiological changes, multiple medications, and coexisting medical conditions. The findings reveal that severe hyponatremia is prevalent in this population, especially in the context of hypovolemia, with generalized weakness and disorientation being common clinical presentations. The study underscores the importance of individualized patient assessments, as the etiological factors of hyponatremia vary significantly, with post-operative conditions and gastroenteritis being more common in this cohort compared to other studies where cardiac and hepatic conditions were predominant.

Despite the relatively lower prevalence of comorbidities in this study, the need for routine monitoring and a comprehensive, tailored approach to treatment is paramount to improving outcomes in elderly patients with hyponatremia. The limitations identified, such as the sample size and cross-sectional design, suggest that further research, including longitudinal studies, is necessary to better understand the long-term impact of hyponatremia and to refine management strategies for this vulnerable population. The observed variation in underlying causes across different studies, including those by Saeed et al. and Baji et al., reinforces the need for personalized treatment plans that address the specific etiologies of hyponatremia in elderly patients.

## Appendices

Case proforma

Patient details

Name:

Age:

Sex:

Date of admission:

Date of discharge:

Primary diagnosis:

Symptoms

Symptoms: Present /Absent

Description of symptoms:

Duration of symptoms:

Diet and fluid intake

Fluid intake:

Diet habits:

Drug history

Co-morbidities

Clinical findings

Pulse rate:

Blood pressure:

Volume status at the time of admission: Hypovolemic/Euvolemic/Hypervolemic

Oedema: Yes/No

Ascites: Yes/No

Biochemical parameters

Serum sodium:

Serum potassium:

Serum chloride:

Serum osmolality:

Urine osmolality:

Urine sodium:

Calculated sodium deficit:

Random cortisol level:

Thyroid function test:

Fasting lipid profile:

Outcome: Recovered/ DAMA discharge / death
